# A Fatal Case of Severe Leg Edema and Necrotic Ulcers in a 14-Year-Old Boy

**DOI:** 10.7759/cureus.70467

**Published:** 2024-09-29

**Authors:** Camilo Mariano-Rodriguez, Mario Magana

**Affiliations:** 1 Service of Dermatology, General Hospital of Mexico "Dr. Eduardo Liceaga", Mexico City, MEX

**Keywords:** hydroa vacciniforme-like lymphoma, hematology and oncology, hematology-oncology, pediatric case, epstein- barr virus, cutaneous t cell lymphoma

## Abstract

A 14-year-old boy presented with edema, ulcers, tenderness, and progressive functional limitation of both legs, first diagnosed as Henoch-Schonlein vasculitis. Then, he underwent one inguinal lymph node excision and two skin biopsies which reported an angiocentric lymphoproliferative process, EBER (Epstein-Barr virus-encoded small RNA) positive, consistent with hydroa vacciniforme-like lymphoproliferative disorder (HVLPD); after eight weeks, his face presented with edema and ulcers, characteristic of the original patients described with HVLPD. The patient's parents refused treatment and took him back home, and he died a few months later. Our case study highlights an atypical localization of the disease, as it initially presented in the lower extremities rather than the face, posing a diagnostic challenge that was ultimately resolved through biopsy.

## Introduction

The hydroa vacciniforme-like lymphoproliferative disorder (HVLPD) is a rare and aggressive cutaneous T-cell lymphoma; the pathogenesis of this disease is crucial for improving diagnostic accuracy and developing more effective treatments. It has been proposed that chronic Epstein-Barr virus (EBV) infection, along with UV radiation, plays a crucial role in stimulating the development of T cells. The most common clinical topography includes the face, neck, and upper extremities [1,2]. The morphological presentation consists of confluent papulovesicular lesions forming plaques, which progress to ulcers and depressed scars. The disease is also characterized by notable edema in the facial region. Diagnosis is based on histopathology and immunohistochemistry, and it is essential to demonstrate EBV infection. Current therapeutic strategies involve nucleoside analog antiviral agents, corticosteroids, chemotherapy regimens, immunoglobulins, anti-CD20 therapy, hematopoietic stem cell transplantation, and several other treatments that are still under investigation [3]. However, the response to these therapies is variable and generally poor, resulting in an overall unfavorable prognosis.

This article was previously posted to the Research Square preprint server on 07 May 2024.

## Case presentation

A 14-year-old boy was admitted with a seven-month history of diffuse edema and erythema in his right leg following an unspecified insect bite. Five months later, he developed a fever, blisters, and edema in both legs. He sought medical attention at his hometown clinical center, where he was treated with various antibiotics, including amikacin, ceftriaxone, imipenem, and chlorthalidone, as well as intramuscular dexamethasone for one month, but showed little to no response. Upon admission, he was in a wheelchair due to functional limitations in both legs. He had no prior medical history, and his immunizations were up to date.

**Figure 1:**
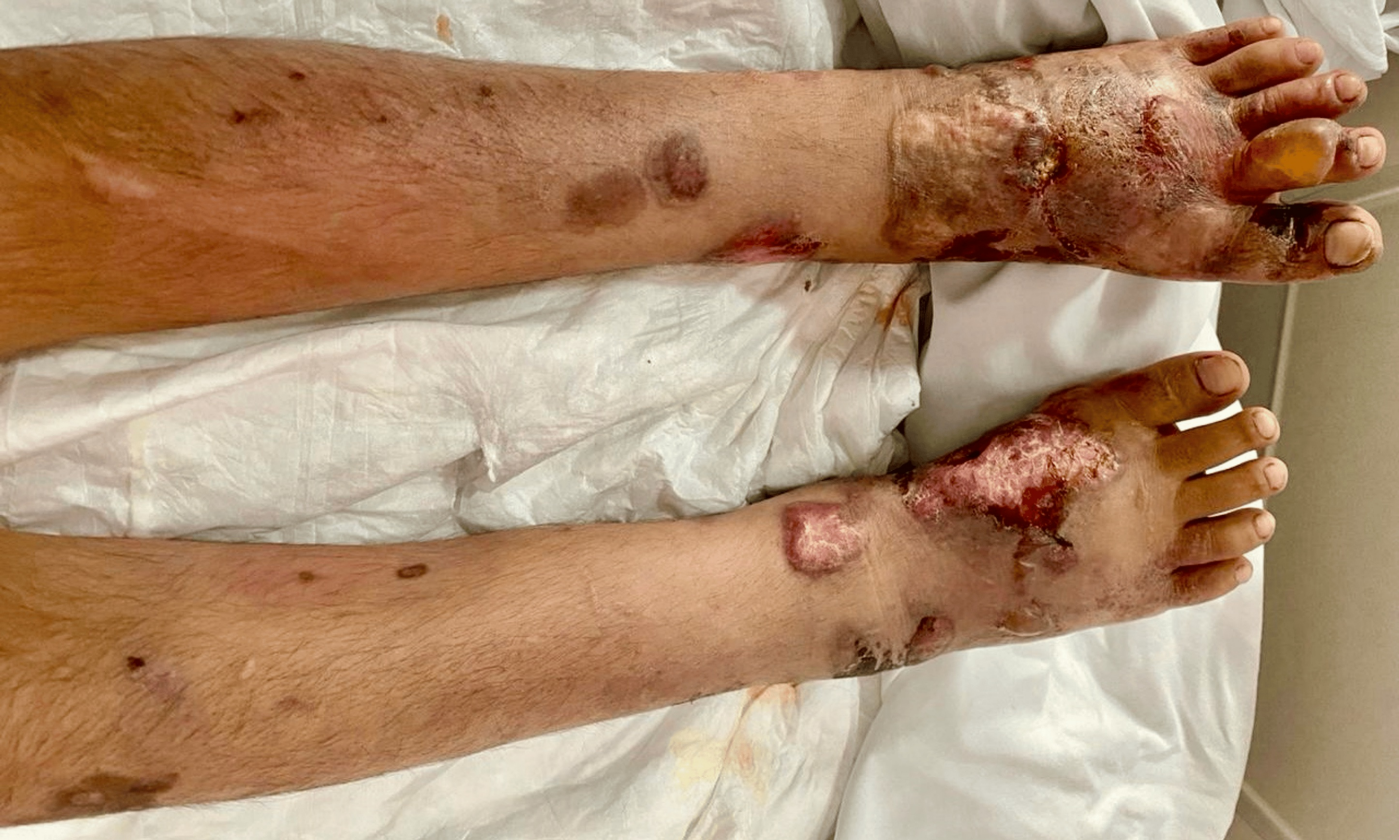
Legs and feet with edema, infiltrated erythematoviolaceous plaques, blisters, and necrotic ulcers.

On physical examination, he appeared pale and weak with a temperature of 37.1°C. Examination of the neck revealed cervical adenopathy and his abdomen showed hepatosplenomegaly and inguinal adenopathy. In the pretibial region of both legs and the dorsum of both feet, there was edema, infiltrated erythematoviolaceous plaques, blisters, and necrotic ulcers (Figure 1), but distal pulses were preserved. An abdominal and pelvic computed tomography (CT) scan revealed hepatosplenomegaly, bilateral inguinal adenopathy, and retroperitoneal lymph nodes (we do not have the images from the study).

He was initially diagnosed with leukocytoclastic vasculitis (Henoch-Schoenlein purpura) and received three days of systemic steroids, but he did not respond to the treatment. Consequently, he underwent an excision of an inguinal lymph node and two skin biopsies, one from each leg.

**Figure 2:**
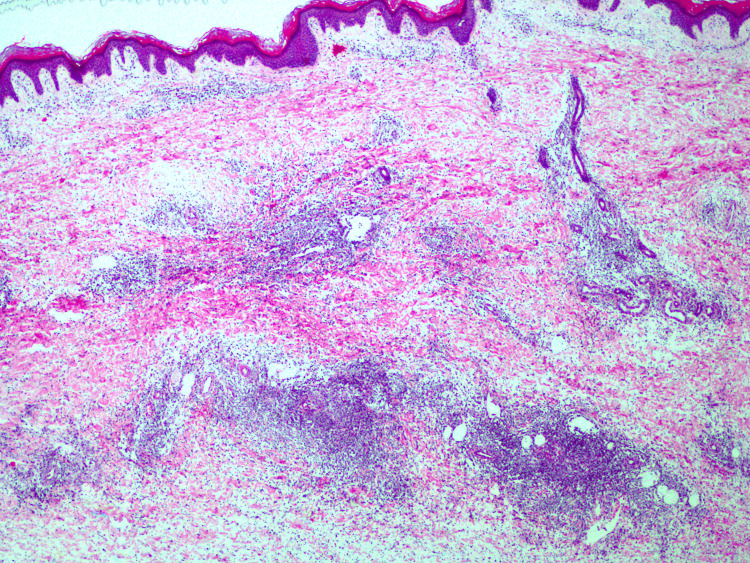
A skin biopsy from the left leg shows evident edema, with superficial, mild, and deep lymphocytic infiltration. The angiocentric and angiodestructive infiltrates are evident.

The biopsy results showed edema of the dermis and hypodermis with a dense superficial and deep lymphocytic infiltrate. Most of the lymphocytes were atypical, primarily small, but there were also medium and large-sized cells, as well as the involvement of some blood vessels by the infiltrate (Figure 2).

**Figure 3:**
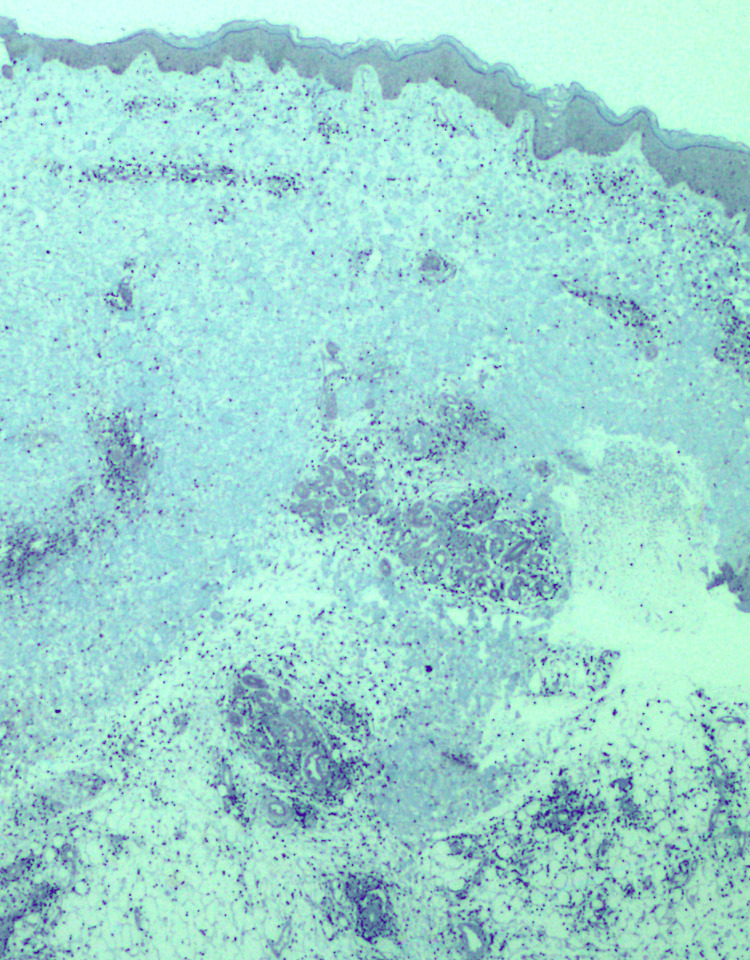
In situ hybridization for Epstein-Barr virus-encoded small RNAs shows clusters of positive cells.

Immunohistochemistry revealed that most of the infiltrate was CD3+, CD4 was negative, CD8 was positive in 80% of the cells, CD30 was positive in 10% of the infiltrate, CD56 was negative, and EBV-encoded small RNAs were positive in most of the cells (Figure 3).

These findings were interpreted as an atypical lymphoid infiltrate that was CD8 positive, leading to a final diagnosis of a lymphoproliferative process consistent with HVLPD.

**Figure 4:**
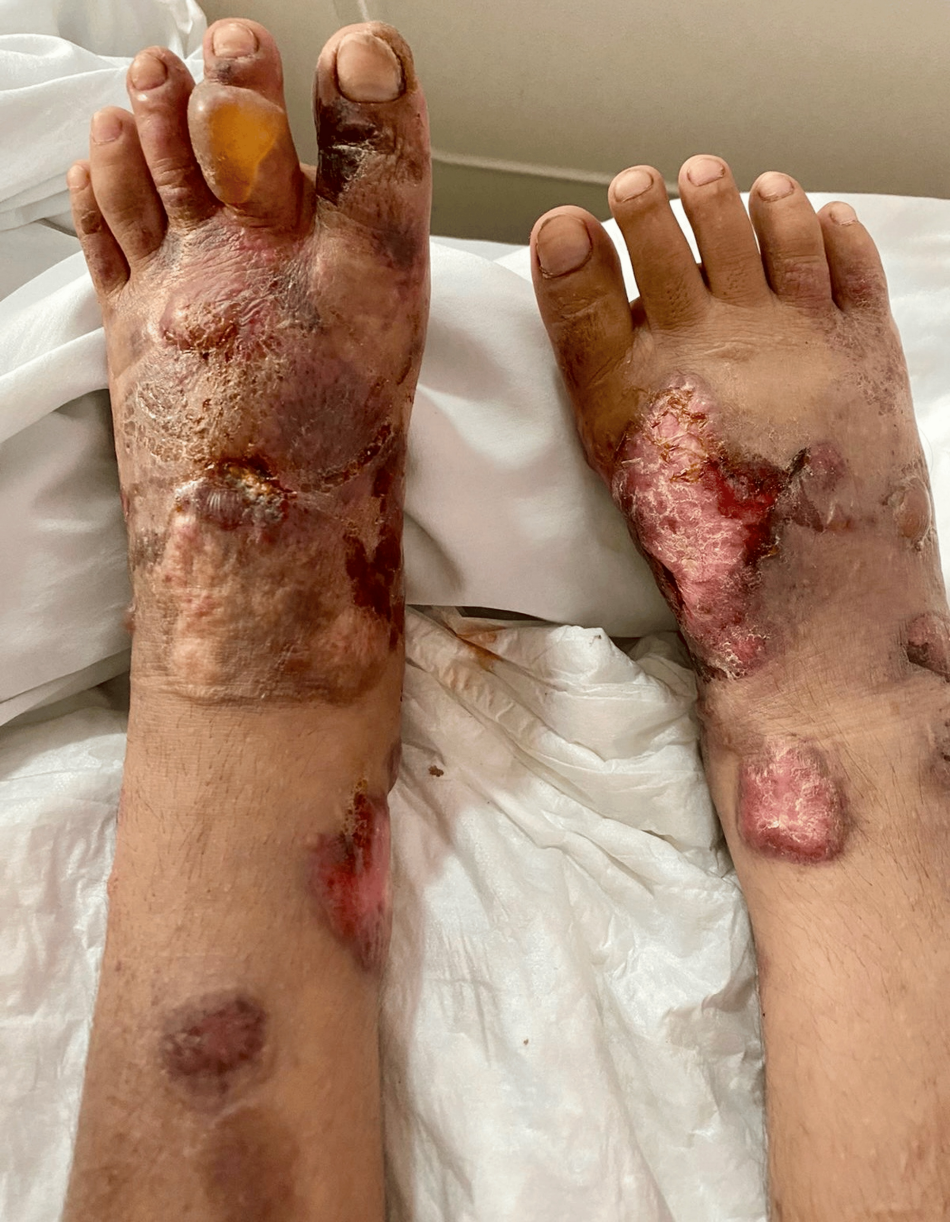
Dorsum of both feet shows edema and necrotic areas.

Following these results, the case was discussed with the hemato-oncology team, who proposed CHOP (cyclophosphamide, doxorubicin, vincristine, and prednisone) therapy. However, the patient's parents declined this treatment and requested a voluntary discharge. Eight weeks later, his last physical examination revealed edema, blisters, and crusts on his face and both legs (Figure 4). He died a few months later in his hometown.

## Discussion

HVLPD is a cutaneous T-cell lymphoma caused by EBV, characterized by a poor prognosis. Its clinical features are distinct, involving edema and vesicular lesions that progress to bullae and later to atrophic or “varioliform” scars, resembling hydroa vacciniforme (HV), a rare photosensitivity disorder. Diagnosing HVLPD presents a significant clinical challenge due to its resemblance to other dermatological conditions, making it difficult to differentiate based on clinical presentation alone. Notably, HVLPD was historically confused histologically with inflammatory diseases like HV or cicatrizing panniculitis with vasculitis, which could predispose patients to malignancy [1-4]. Therefore, it is crucial to perform a diagnostic biopsy, as histopathological examination and immunohistochemistry are essential for confirming the diagnosis and demonstrating EBV infection. Without a biopsy, an accurate diagnosis and subsequent appropriate management may be delayed.

HVLPD was first described in 1998 in four Mexican children, initially named "angiocentric cutaneous T-cell lymphoma of childhood" or "hydroa-like lymphoma associated with EBV" [1]. Earlier studies by Oono in Japan [2] reported only an association between HV and non-Hodgkin lymphoma. In 2002, Barrionuevo et al. demonstrated monoclonality in a series of 16 patients from Peru [5]. Subsequently, the World Health Organization recognized HVLPD as an EBV-driven lymphoproliferative disorder and renamed it HVLPD [6-9].

HVLPD is predominantly diagnosed in female children and adolescents from East Asia and Latin America and is rare in other demographics, which may contribute to a worse prognosis in these populations [8-10].

Etiopathogenesis

The etiology of HVLPD remains uncertain. However, the presence of EBV in skin samples, positive immunohistochemical staining, and serological evidence from patients strongly suggest a link between the virus and the disease. The most widely accepted hypothesis for its pathogenesis involves chronic EBV infection combined with UV radiation as triggers. This interaction promotes the release of antimicrobial peptides from the innate immune response, stimulates γδ T cells, and inhibits effector T cells [11].

EBV, a gamma herpesvirus with double-stranded DNA, is highly contagious and infects nearly all children. The virus enters through the oropharynx, infecting cells (mainly B cells, NK cells, and T cells) which are subsequently lysed, releasing virions. These infected cells are cleared by CD8+ cytotoxic cells. EBV then remains latent in memory B cells; in HVLPD, these cells do not proliferate, so the viral load does not increase. Reactivation, triggered by immunosuppression, allows these latent cells to proliferate, leading to clonal expansion and increasing the risk of mutations [12].

It is unclear whether EBV acts as a causative agent, a cofactor, or simply a coincidental infection in HVLPD. Some evidence suggests that latent membrane protein 1 (LMP-1) promotes lymphomagenesis by blocking apoptosis and inducing cellular proliferation. Similarly, Epstein-Barr nuclear antigen 1 (EBNA-1) induces translocations, and EBNA-2 promotes cellular proliferation. The presence of EBNA-1 and LMP-1 in HVLPD indicates type II latency, which may explain the association between EBV and HVLPD [12].

Cohen et al. proposed that HVLPD severity is influenced by genetic and environmental factors. In a study of 16 HVLPD patients at the National Institutes of Health Clinical Center, 10 were Caucasian, four were Hispanic, and two were Asian. Only one Caucasian patient developed systemic EBV disease, whereas five of six non-Caucasian patients did. Non-Caucasian patients also had higher levels of viral DNA, lower NK cells, and more T-cell clones in the blood. Over several years of follow-up, four non-Caucasian patients required hematopoietic stem cell transplantation, whereas none of the Caucasian patients did. These findings suggest that non-Caucasian patients with HVLPD are at a higher risk of developing systemic EBV disease and have a worse prognosis than Caucasian patients [13].

Clinical and histopathological findings

HVLPD has been misdiagnosed and misclassified for many years. However, recent publications have clarified its clinical and microscopic characteristics, aiding in accurate diagnosis [6,14]. The classic clinical presentation includes facial edema, and recurrent papulovesicular and necrotic eruptions on the face, neck, and extremities. EBV has been detected in the skin tissues of all patients studied [14]. Histopathologically, HVLPD features dense atypical lymphocytic infiltrates involving the dermis and subcutaneous fat, often displaying angiocentric and angiodestructive patterns. These large pleomorphic cells are usually positive for CD3, CD45, CD45Ro, CD8, and CD30, although in some cases, these markers may be negative [15-18].

The disease course is typically aggressive, with progression to involve lymph nodes, bone marrow, liver, spleen, lungs, kidneys, and the central nervous system. There is no standard treatment regimen, but various therapies, including chemotherapy, radiotherapy, and immunomodulatory therapies, have been reported [6]. Most patients succumb to disease complications within an average of 5.3 months after the initial diagnosis [19,20].

## Conclusions

HVLPD is a poorly understood disease, and its treatment options remain limited. Therefore, in cases of clinical suspicion, it is crucial to perform an early biopsy for prompt and accurate diagnosis. Despite this, the prognosis for most patients is unfavorable. The atypical presentation in this case broadens our understanding of the potential morphological and topographical variations of this pathology. In this patient, EBV infection was confirmed through Epstein-Barr virus-encoded small RNA (EBER) in situ hybridization. Additionally, the patient's hometown is a region with a predominantly tropical climate, and both have been proposed as possible contributors to the development of HVLPD. Our study faced limitations, including scarce resources at our hospital and the absence of electronic medical records, which restricted our ability to document imaging and molecular studies thoroughly. Ultimately, this case not only underscores the aggressive and poor prognosis associated with HVLPD but also highlights the increased risk for non-Caucasian patients in developing systemic disease. Further research is urgently needed to explore and develop more effective treatments for HVLPD that can provide better responses to the disease and potentially improve patient prognosis.
